# In Vitro fertilization (IVF) treatments in Maccabi Healthcare Services 2007-2014

**DOI:** 10.1186/s13584-016-0072-9

**Published:** 2016-04-08

**Authors:** Shahar Kol, Lucia Bergovoy Yellin, Yaakov Segal, Avi Porath

**Affiliations:** Maccabi Healthcare Services, Tel Aviv, Israel; Department of Health Services Research at Chief Physician Office of Maccabi Healthcare Services, Tel Aviv, Israel

**Keywords:** In-vitro fertilization, Assisted reproductive technology, Maccabi Healthcare Services, Israel, Infertility registry

## Abstract

**Background:**

Israel reports the world’s highest IVF cycles per capita. However, clinical outcome data of these treatments are scarce. In a previous publication, we summarized IVF results among Maccabi Healthcare Services members for the years 2007-2010. The main findings included an increase in mean patients’ age over the period studied, a 50 % increase in cycle numbers during this time, and a decrease in success rate (live birth) from 18.8 % in 2007 to 14.8 % in 2010. The purpose of the current publication is to summarize IVF outcome for the years 2011-2014, and to explore possible changes in the trends we reported previously.

**Methods:**

IVF and live births data were collected from Maccabi Healthcare Services’ fertility treatments registry. Analyses were conducted by treatment year and patients’ age at the initiation of treatment cycles. Autologous cycles, were included (ovum donation cycles and frozen-thaw cycles were excluded). A successful cycle was defined if a live birth was recorded within 10 months of its initiation.

**Results:**

In accordance with previous data for the years 2007-2010, mean patients’ age continued to rise (from 36.2 in 2011 to 37.1 in 2014). In contrast to previous years, during which a continued increase in treatment cycles was recorded, we found that treatment number decreased from a peak of 9,751 in 2011 to 8,623 in 2014. Contrary to that trend, the number of patients over 40 years of age increased from 3,204 in 2011 to 3,648 in 2014. Success rate fluctuated between 14.4 % in 2014 to 16.4 % in 2013. The majority (78 %) of treatment cycles were conducted in four private medical centers.

**Conclusions:**

The decrease in treatment cycles in recent years notwithstanding, Israel is still leading the world with IVF treatments relative to population. Success rate is relatively low compared to international data. Given the steady increase in patients’ mean age, and particularly, the increase in patients over 40 years of age, we maintain that the low success rate reflects a growing number of treatments that *a priori* have a low chance of success.

## Background

The number of IVF cycles performed in Israel (relative to population size) is the highest in the world [1]. The main reason is the unprecedented Israeli IVF health basket, which provides practically unlimited IVF treatments to eligible infertility patients who are under 45 years old and have no more than two children, including single mothers [2]. While this policy was challenged by professional organizations, it seems that for many years politicians were reluctant to limit IVF treatments. Moreover, the Ministry of Health as the regulatory body stated in 2014 that given medical considerations, IVF treatment could be regarded as first line of treatment for patients over 39 years of age [3].. When professional considerations to stop treatments before the 45 year age limit were challenged in court [4], the judge decided in favor of the patient, stating that the maximal age for treatment (45) was decided based on medical considerations and the current medical literature. Of note, the pertinent professional body (The Israel Fertility Association) recommended lowering the upper age limit for IVF treatments.

The relative low “out of pocket” cost, and the high availability of IVF services in Israel [4] contributed to the popularity of IVF treatments. Currently, IVF services are available in all Israeli public medical centers (excluding Zefat, the 3 Nazareth medical centers and Eilat), in addition to four IVF units in private medical centers (Elisha, HMC, Assuta Tel Aviv and Assuta Rishon LeZion).

IVF activity is monitored by a long list of national and international organizations, most notably the Society for Assisted Reproductive Technologies (SART) [5] and the Centers for Disease Control and Prevention (CDC) (http://www.cdc.gov/art/reports/) in the United States, and the European Society for Human Reproduction and Embryology (ESHRE) in Europe [6]. Currently, an effort to establish an Israeli IVF registry is underway, though periodic comprehensive reports are not yet available. Professor Liat Lerner-Geva presented preliminary data on clinical pregnancy rate at the 2015 annual meeting of the Israel Fertility Association. The Ministry of Health publishes limited retrospective annual reports [7]. According to these reports available from 2000 to 2013, live birth rate per treatment cycles ranges between 14.9-17.2 %. These results fall significantly short of the reported live birth rate per treatment cycles as published by the above registries.

Previously, we summarized IVF results among Maccabi members for the years 2007-2010 [8]. The main findings included an increase in mean patients’ age over the period examined, a 50 % increase in cycle numbers during this period, and a decrease in success rate (live birth) from 18.8 % in 2007 to 14.8 % in 2010. The purpose of the current communication is to summarize the IVF outcomes for the years 2011-2014, to explore possible changes in the trends we reported previously, while deepening the analysis and exploring the policy implications of the findings.

## Methods

Maccabi Healthcare Services is the second largest Health Maintenance Organization (HMO) in Israel covering 433,711 women in fertility ages (15-45) according to the National Insurance Institute report of November 2014 [9], or 25.7 % of the total fertility age population (1,687,873 women).

In an attempt to gain further insight on IVF activity in Israel, we analyzed data generated from the Maccabi Healthcare Services fertility treatment registry. The responsible HMO reimburses all IVF treatments for Israeli citizens (in public and private medical centers alike); therefore, reliable information is gathered on the number of cycles performed in all IVF units.

All IVF treatments of Maccabi members are routinely registered as part of the reimbursement system that serves the financial infrastructure to all treatments performed. Live birth is reimbursed by the National Insurance Institute of Israel, and therefore is not directly reported to the pertinent HMO. However, as a default, the newborn is registered to his/her mother’s HMO, generating a significant financial movement for the HMO (one more member). A cross-match between these two financial movements (paying the medical center for IVF and adding a new member to the HMO) can yield a good estimate (though not perfect) of live birth rate post IVF, if a live birth occurred within 10 months from the IVF treatment.

In the current publication we included “fresh” cycles, defined as ovarian stimulation + oocyte retrieval (“phase 1”) followed by fertilization and embryo transfer 2 – 6 days after oocyte retrieval (“phase 2”). We collected data on patients’ age on the day of IVF treatment, the specific medical center where treatment was given, and number of cycles performed in each medical center.

Data for 2011-2014 were collected and summarized in late September 2015; therefore, we assume that all pregnancies achieved in 2014 have ended by that time.

## Results

### Treatments and live birth rate

The 8 years surveyed can be divided into 2 periods: A steady increase in the number of treatments from 2007 (6,242 treatments) to a peak in 2011 (9,751 treatments), and a moderate decrease in treatments thereafter. Live birth rate decreased from 18.9 % in 2007 to 14.4 % in 2014 (Table 1).

**Table 1:** Number of IVF treatments performed, number of live births achieved from these treatments, and success rate by year (2007-2014)

Year	Number of patients	Number of treatments	Number of live births	Cycles/patient	Live birth/cycle	Live birth/patient
2007	4,061	6,242	1,182	1.54	18.9 %	29.1 %
2008	4,410	7,041	1,295	1.60	18.4 %	29.4 %
2009	4,867	8,336	1,356	1.71	16.3 %	27.9 %
2010	5,282	9,297	1,384	1.76	14.9 %	26.2 %
2011	5,479	9,751	1,429	1.78	14.7 %	27.9 %
2012	5,375	9,314	1,438	1.73	15.4 %	26.7 %
2013	5,360	8,455	1,386	1.58	16.4 %	25.9 %
2014	5,577	8,623	1,238	1.55	14.4 %	22.2 %
Total	40,411	67,059	10,708	1.66	16.0 %	26.5 %

### Treatments and live birth rate according to patients’ age groups

**Fig. 1:**
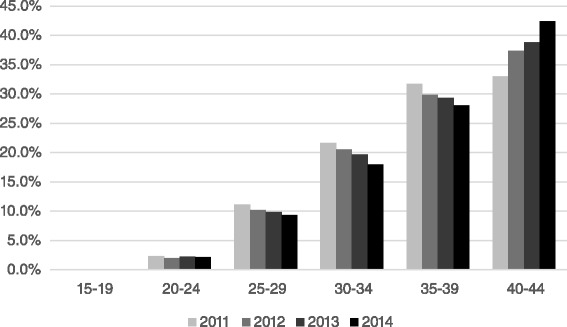
Age distribution of cycles by year, 2011-2014

From 2011 to 2014, a steady decrease was noted in the treatments performed in the younger age groups (25-39), with a parallel steady increase in the 40-45 age group. A sharp decrease in live birth was noted for patients over 40 years of age (Table 2, Fig. 1).

**Table 2:** Number of IVF treatments and success rate by year (2011-2014) and age group

Year	Age group	Number of treatments	Number of live births	Success rate [%]	Distribution by age
2011	15-19	4	0	0	0.0 %
	20-24	226	56	24.8	2.3 %
	25-29	1,086	277	25.5	11.2 %
	30-34	2,104	449	21.3	21.7 %
	35-39	3,082	479	15.5	31.8 %
	40-45	3,204	168	5.2	33.0 %
2012	15-19	7	2	28.6	0.1 %
	20-24	187	49	26.2	2.0 %
	25-29	947	273	28.8	10.2 %
	30-34	1,905	437	22.9	20.5 %
	35-39	2,772	468	16.9	29.9 %
	40-45	3,466	209	6.0	37.3 %
2013	15-19	4	0	0.0	0.0 %
	20-24	188	45	23.9	2.2 %
	25-29	832	235	28.2	9.9 %
	30-34	1,657	452	27.3	19.7 %
	35-39	2,470	425	17.2	29.3 %
	40-45	3,271	228	7.0	38.8 %
2014	15-19	4	0	0.0	0.0 %
	20-24	184	52	28.3	2.1 %
	25-29	804	203	25.2	9.4 %
	30-34	1,547	380	24.6	18.0 %
	35-39	2,411	386	16.0	28.0 %
	40-45	3,648	217	5.9	42.4 %

### Mean patients’ age

A steady increase in patients’ mean age was documented from 2007 (35.1) to 2014 (37.1).

### Mean patients’ age by type of medical center

From 2007 to 2014, 16,004 and 51,055 treatments were performed in public and private medical centers, respectively. Patients’ mean age was 35.5 and 36.5 in public and private medical centers, respectively (Table 3).

**Table 3:** Mean age of IVF patients by medical center type (public/private), 2011-2014

Medical center type	Mean age	Number of treatments
Public	35.46	16,004
Private	36.50	51,055
Total	36.25	67,059

### Detailed outcome for the 40-45 age group

In all the years surveyed, a steady decrease in live birth rate was noted from age 40 to 44. Two thousand two hundred and three treatments were performed in women 44 years of age from 2011 to 2014, yielding 35 live births (1.6 %). (Table 4).

**Table 4:** Number of IVF treatments and success rates for women ≥40 year old age group, by year (2011-204) and age

Year	Age	Number of treatments	Number of live births	Success rate [%]
2011	Total	3,204	168	5.2
	40	660	68	10.3
	41	736	38	5.2
	42	716	31	4.3
	43	555	21	3.8
	44	537	10	1.9
2012	Total	3,466	209	6.0
	40	716	89	12.4
	41	789	52	6.6
	42	741	37	5.0
	43	649	21	3.2
	44	571	10	1.8
2013	Total	3,271	228	7.0
	40	635	74	11.7
	41	757	72	9.5
	42	736	57	7.7
	43	599	20	3.3
	44	544	5	0.9
2014	Total	3,648	217	5.9
	40	781	77	9.9
	41	855	59	6.9
	42	759	43	5.7
	43	702	28	4.0
	44	551	10	1.8

### Live birth rate by medical center

Since outcome of a small number of treatments has limited statistical significance, we decided to include medical centers with >100 treatments per year in the analysis. There are significant changes in success rate between medical centers, and significant changes within the same medical center during the 4 years surveyed (Tables 5 and 6).

**Table 5:** Number of IVF treatments and success rate, by year (2011-2012) and medical center (only medical centers with ≥100 cycles per given year were included)

Year	Medical center^a^	Number of treatments	Number of live births	Success [%]
2011	Total	9,751	1,429	14.7
	A	914	93	10.2
	B	561	40	7.1
	C	673	114	16.9
	D	1,958	387	19.8
	E	3,908	502	12.8
	F	223	49	22.0
	G	120	24	20.0
	H	307	48	15.6
	I	161	40	24.8
	J	303	18	5.9
	K	170	37	21.8
2012	Total	9,314	1,438	15.4
	A	889	101	11.4
	B	420	44	10.5
	C	637	112	17.6
	D	1,988	371	18.7
	E	3,574	522	14.6
	F	248	42	16.9
	G	109	25	22.9
	H	321	48	15.0
	I	152	34	22.4
	J	346	37	10.7
	K	189	28	14.8

^a^Medical center: in order to protect the confidential information, the names of the medical centers were recoded into random letters

**Table 6:** Number of IVF treatments and success rate, by year (2013-2014) and medical center (only medical centers with ≥100 cycles per given year were included)

Year	Medical center^a^	Number of treatments	Number of live births	Success [%]
2013	Total	8,455	1,386	16.4
	A	809	123	15.2
	B	250	29	11.6
	C	448	94	21.0
	D	1,851	347	18.7
	E	3,262	505	15.5
	F	231	40	17.3
	G	105	21	20.0
	H	291	53	18.2
	I	156	27	17.3
	J	290	40	13.8
	K	184	21	11.4
	L	149	22	14.8
2014	Total	8,623	1,238	14.4
	A	1,136	176	15.5
	B	268	27	10.1
	C	459	75	16.3
	D	1,957	352	18.0
	E	2,877	347	12.1
	F	187	37	19.8
	G	115	18	15.7
	H	195	26	13.3
	I	178	36	20.2
	J	312	39	12.5
	K	262	26	9.9
	L	188	33	17.6

^a^Medical center: in order to protect the confidential information, the names of the medical centers were recoded into random letters

### Treatment distribution between public and private medical centers

**Fig. 2:**
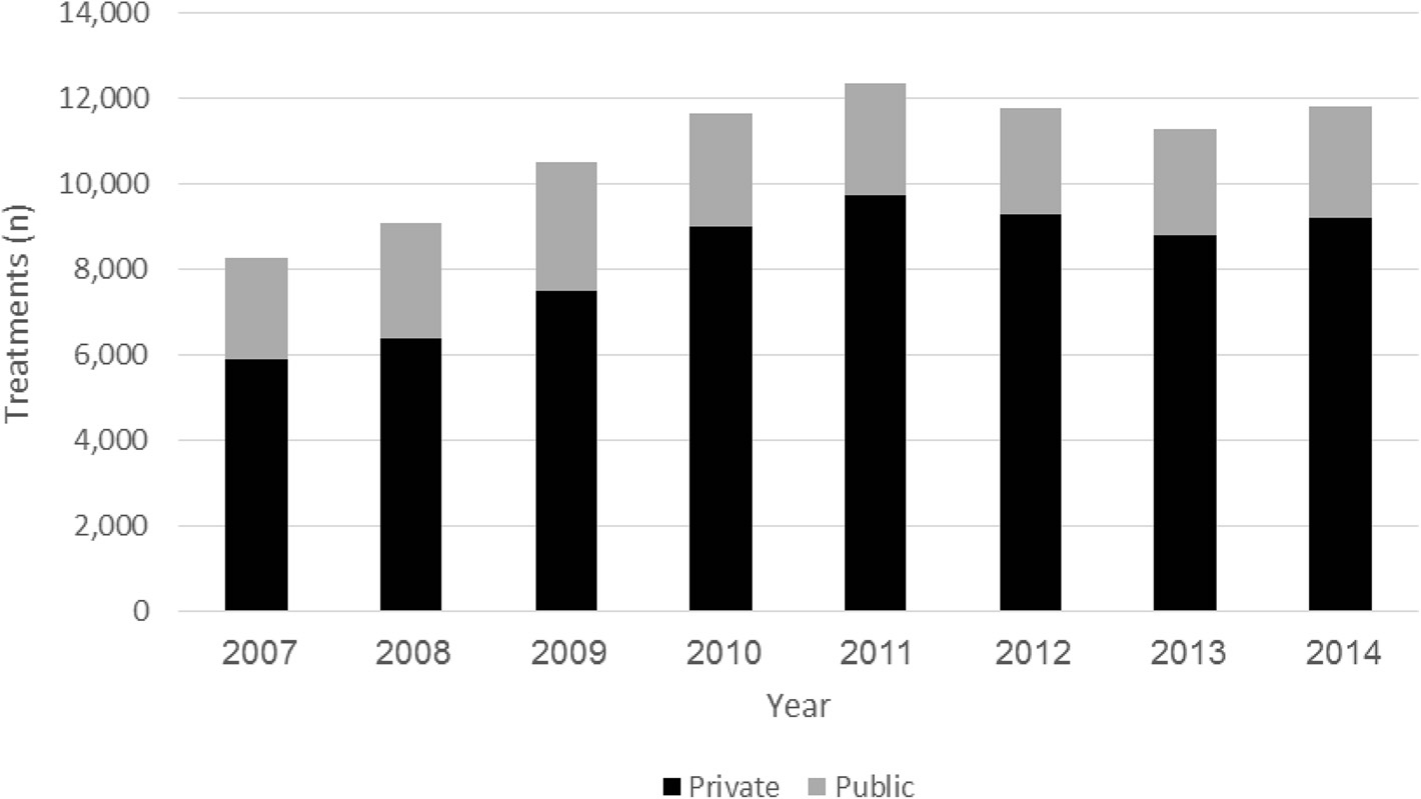
IVF Treatments distribution between public and private medical centers, by year for 2007-2014

In 2007, 2,401 and 5,896 treatments were performed in public and private medical centers, respectively. Treatments in public medical centers increased marginally from 2007 to 2014 (2,618 treatments in 2014, a 9 % increase). Treatments in private medical centers increased significantly from 2007 to 2014 (9,211 treatments in 2014, a 56 % increase) (Fig. 2).

## Discussion and conclusions

In the current publication, we update a previous report [8], and present an eight-year summary of IVF treatments in Maccabi Healthcare Services. Since Maccabi covers approximately 25 % of the population in Israel, the data herein reliably represent the total IVF activity in Israel. We report a modest decrease in treatment cycles in recent years. The Israeli success rate is low compared to international data (http://www.cdc.gov/art/reports/) [5, 6]. Of note is a steady increase in patients’ mean age, and particularly, an increase in patients greater than 40 years old. While both live birth per cycle and per patient show a steady decline from 2007 through 2014, there is a marked decrease in the number of cycles per patient from the peak in 2011, suggesting possible changes in practice.

The health policy decisions that culminated in an unprecedented coverage of IVF treatments in Israel reflect societal and political considerations, as opposed to pure professional, evidence-based considerations. Naturally, these health policy decisions have a significant price tag. Updated in 01-September-2015, according to the Ministry of Health, an IVF treatment reimbursement costs 12,000 NIS [10]. We report herein that 2,203 treatments (26,436,000 NIS) were performed in women 44 years of age from 2011 to 2014, yielding 35 live births. The cost of a single live birth in that age group was 755,314 NIS (not including fertility medications supplied by Maccabi).

Previous efforts of professional organizations (most notably The Israel Fertility Association – IFA) to divert resources in a more cost effective way (i.e. ovum donation) have failed. Moreover, previous professionally based guidelines by the Ministry of Health itself to minimize futile treatments were not implemented. Although in 1999, such guidelines were adopted (http://www.ayala.org.il/?CategoryID=239&ArticleID=77) based on recommendations made by a professional committee, nominated by the Minister of Health, the guidelines are not currently implemented. Moreover, a timely update was not published. Given current IVF health policy, the practice of IVF shifted from a medical treatment bearing indications and contra-indications, into a social, age-related right [11]. This shift was fully endorsed by the legal system [12].

Economic costs of IVF treatments have a significant impact on the Israeli HMO’s finances. A survey conducted in Maccabi in 2006 found that 5.4 % of health expenditures for women related to fertility treatments, more than was spent on diabetes (3.5 %) and comparable to expenditures on cardiovascular diseases (5.9 %) [13].

Naturally, IVF health policy raises significant ethical considerations. The Israel Fertility Association ethical committee published its position regarding futile IVF treatments in February 2015 [14]. The committee defined a “futile treatment” as a treatment in which the chance for live birth is <1 %, and strongly denounced performing such treatment. Yet, experience shows that these considerations are defeated when challenged.

In the current publication, we detail the live birth rate achieved in different medical centers. These data must be interpreted with caution given the lack of pertinent individual clinical information i.e. indications, number of oocytes retrieved, number of embryos obtained and their quality, previous IVF failures, usage of specific technologies (pre-implantation genetic diagnosis, testicular sperm extraction, in-vitro maturation etc.). We speculate that units differ significantly as to their patients’ treatment prognosis. In addition, some units impose a 44-age limit, though, as mentioned above, the IVF national health basket covers women until 45 years of age. Notably, the number of treatments performed in “Clalit” public medical centers is low; therefore, these units are under-represented in our data.

According to the Ministry of Health, 39,174 IVF cycles were performed in 2013 [7], with a live birth rate of 15.7 % (comparable to our data of 16.4 % for that year). According to the CDC (http://www.cdc.gov/art/reports/) 163,212 cycles were performed in the US in the same year with a live birth rate of 33 %. In our opinion, this comparison highlights the fundamental problem that erodes IVF clinical outcomes in Israel: Too many cycles are performed despite a very slim chance of success.

IVF health-related policy is a subject of public and professional debate, in which the question of resource allocation should be thoroughly and openly discussed. As pointed out previously [15], the existing policy of assisted reproduction in Israel, that is, of unlimited rounds with IVF, should be further questioned and assessed. Possible conclusions of such an assessment may be that IVF treatments should cease before 45 years of age, and/or limiting treatment number for an individual patient.
